# Alginate Gel-Based Carriers for Encapsulation of Carotenoids: On Challenges and Applications

**DOI:** 10.3390/gels9080620

**Published:** 2023-08-01

**Authors:** Milan Milivojević, Aleksandra Popović, Ivana Pajić-Lijaković, Ivan Šoštarić, Stefan Kolašinac, Zora Dajić Stevanović

**Affiliations:** 1 Faculty of Technology and Metallurgy, University of Belgrade, Karnegijeva 4, 11120 Belgrade, Serbia; 2 Faculty of Agriculture, University of Belgrade, Nemanjina 6, 11080 Belgrade, Serbia

**Keywords:** alginate gel, carotenoids, food encapsulation, Raman spectroscopy, controlled release, review

## Abstract

Sodium alginate is one of the most interesting and the most investigated and applied biopolymers due to its advantageous properties. Among them, easy, simple, mild, rapid, non-toxic gelation by divalent cations is the most important. In addition, it is abundant, low-cost, eco-friendly, bio-compatible, bio-adhesive, biodegradable, stable, etc. All those properties were systematically considered within this review. Carotenoids are functional components in the human diet with plenty of health benefits. However, their sensitivity to environmental and process stresses, chemical instability, easy oxidation, low water solubility, and bioavailability limit their food and pharmaceutical applications. Encapsulation may help in overcoming these limitations and within this review, the role of alginate-based encapsulation systems in improving the stability and bioavailability of carotenoids is explored. It may be concluded that all alginate-based systems increase carotenoid stability, but only those of micro- and nano-size, as well as emulsion-based, may improve their low bioaccessibility. In addition, the incorporation of other biopolymers may further improve encapsulation system properties. Furthermore, the main techniques for evaluating the encapsulation are briefly considered. This review critically and profoundly explains the role of alginates in improving the encapsulation process of carotenoids, suggesting the best alternatives for those systems. Moreover, it provides a comprehensive cover of recent advances in this field.

## 1. Introduction

Alginates and alginate hydrogels have gained significant attention in various fields within recent decades, such as food technology, pharmacy, biomedical engineering, nanotechnology, biotechnology, and many others [1,2,3,4,5,6,7,8]. The usage of alginates in a wide range of applications has been mostly due to their exceptional gelling, stabilizing, and rheological behavior, along with their remarkable water retention capacity. They are commonly utilized in the form of beads, capsules, fibers, films, and blends with other natural and synthetic polymers [1]. Alginate-based gels are “a first choice” among natural polymers for encapsulation by ionic gelation [3]. The main reason for such large interest and wide applications are preferable properties of alginate gels. Generally, alginates are cheap, abundant, harmless, biocompatible, biodegradable, mucoadhesive, easy, and, in mild conditions gelling biopolymer [1,4,5]. However, while their gelling process is simple, the performance of thus formed alginate networks is strongly affected by the molecular structure of used alginate and the kinetic of the cross-linking process. Therefore, in order to be used as coatings or/and delivery system, one should know how those factors will influence the thickness, porosity, permeability, mechanical strength, and swelling behavior of the formed gel [6]. In addition, the properties of natural alginates may be further tuned either by chemical modifications or by blending with other polymers, such as proteins, starch, chitosan, etc. For the formulation of novel and smart drugs or other active compound delivery systems, with enhanced therapeutic efficacy, better patient compliance, and cost-effectiveness, one of the main tasks is the proper selection of materials and technologies for its production. In order to fulfill these requests, one must have an appropriate understanding of numerous polymer characteristics, such as extraction methodology and sustainable production, chemistry, surface characteristics, rheology, bulk properties, biocompatibility, biodegradability, etc. [9]. Consequently, if someone wants to properly use alginate as a functional gelling agent, that will not only efficiently preserve the stability of the encapsulated bioactive compounds for anticipated time and under specific conditions but also accomplish its sustained/controlled release at a desired location within the human body. Basic knowledge about the chemical and physical properties of the alginate gel, its possible chemical modifications, limitations, as well as behavior in blending with other polymers should be known. In addition, in recent years, awareness about the importance of using eco-friendly and non-toxic materials, as well as production procedures, has emerged, and in this context, the use of alginate gels should gain increased attention [10].

Among various alginate applications, the widest ones are those in food technology and biomedicine. As it is well known, in this industry, dehydration and freezing are two main unit operations used for conservation. However, when raw materials containing some sensitive active compounds, such as carotenoids and others, are subjected to those operations, high losses in composition or/and functionality are usually observed [7]. On the other hand, while carotenoids within fruits and vegetables are easily degraded [8,11], those encapsulated in alginate hydrogel matrices show not only improved chemical stability but also increased bioavailability [12].

In order to functionally encapsulate active compounds, such as carotenoids, it is necessary to adequately choose an encapsulating matrix and to find an appropriate method for encapsulation. To be used for encapsulation of active compounds in the food and pharmaceutical industry, encapsulating material must be in the GRAS (generally recognized as safe) category [13]. In most cases, these are food-grade biopolymers, such as carbohydrates, proteins, lipids, and other biodegradable polymers, that may have nutritional or/and structural functions in humans [14]. Among them, polysaccharides are the most vastly used because they are highly biodegradable and biocompatible, have a low price, and are abundant [2,9,15]. When carotenoids are encapsulated, a proper choice of encapsulating agent is very important [16]. In addition, not only the physicochemical but also rheological properties of encapsulating material should be considered [17]. Some of the most important general characteristics that an encapsulating matrix should have are the following: Easy and strong network forming, low price and abundance, appropriate dissolution, biocompatibility, and biodegradability, mechanical and chemical stability under working and storage conditions, ability to preserve encapsulated materials, capability for targeting and sustained release [14].

Besides the many different encapsulating materials, hydrogels are particularly preferable for food and biomedical applications. The main reasons for this are numerous, but one of the most important is that hydrogels have very adjustable properties as the porosity, swelling, stability, ability to respond to different external stimuli (i.e., pH, temperature, or change in ionic strength), and others that can be controlled by regulating cross-linking density of produced encapsulating gel [9,18]. The methods for gel preparation are based on physical cross-linking (by ionic interactions, hydrogen bonding, complex coacervation, or freeze-thawing), chemical cross-linking (by grafting or using chemical cross-linkers), radiation cross-linking (in paste, solid, or aqueous state) and polymerization technique (by bulk or solution polymerization). The alginate gel beads with entrapped active components have been produced by: (1) Simple extrusion (dripping), (2) extrusion by electrostatic potential, (3) extrusion combined with vibration, spinning, or spray nozzle, (4) extrusion combined with jet cutting or spinning disc, (4) impinging aerosol, (5) emulsification, (6) microfluidic technique, and (7) hybrid microgel technique. Each of those methods has its own advantages and limitations and produces gel carriers with different sizes and properties [5].

Within this review, the main properties of carotenoids, as well as the main problems associated with their use in different food and medical applications, are given and summarized. The importance of carotenoid encapsulation and detailed compilation of different alginate-based systems used for this purpose is also provided with critical consideration of their abilities and limitations. Since appropriate use of alginate needs profound knowledge of its main properties, a detailed description of all important features related to alginate gels is also given. Finally, the main techniques for evaluating the encapsulation are also briefly considered.

## 2. Carotenoids

**Figure 1:**
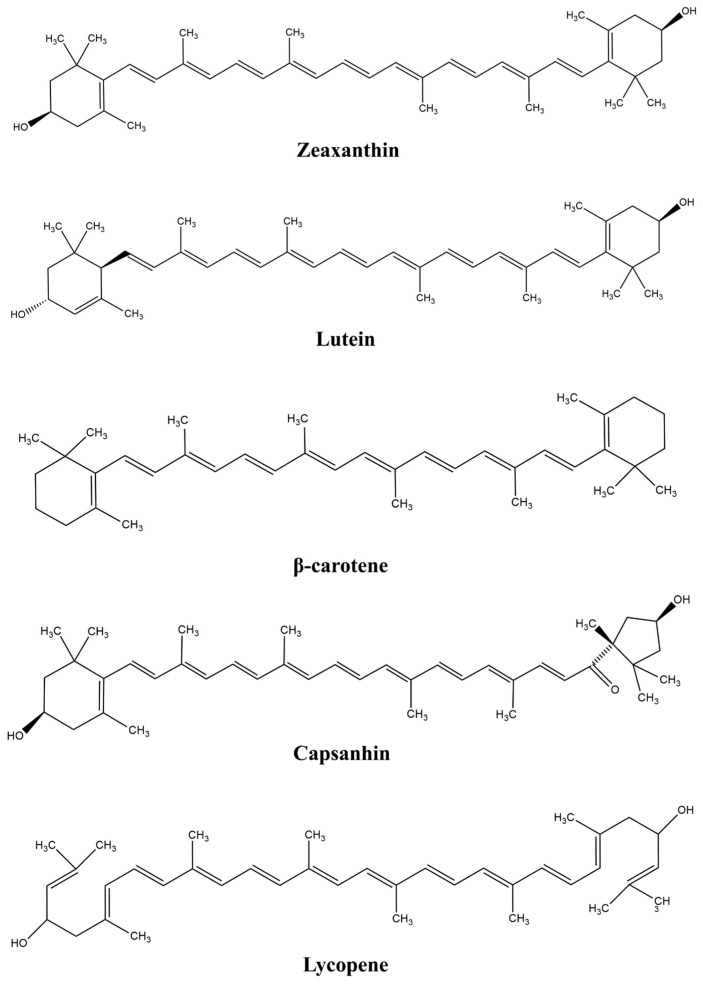
Schematic presentation of the main carotenoid structure.

Carotenoids, a family of terpenoid pigments responsible for the red, orange, and yellow colors of fruits and vegetables, are synthesized by all plants and many microorganisms (bacteria and fungi). They are built of eight isoprene units linked to a skeleton of 40 carbon atoms with conjugated double bonds in a polyene chain. The central carbon chain is mostly composed of different cyclic or acyclic end groups [19]. They may be classified into two main groups, hydrocarbon carotenoids (carotenes) (i.e., α-, β-carotene, lycopene) and oxygenated carotenes (xanthophylls) (i.e., astaxanthin, β-cryptoxanthin, canthaxanthin, capsanthin-capsorubin, fucoxanthin, lutein, zeaxanthin) [20,21]. Xanthophylls contain oxygen in the form of keto, epoxy, aldehyde, or hydroxyl groups. These different structures of carotenoids contribute to a wide range of yellow, red, and orange colors. In metabolic pathways, the first C40 precursor of carotenoids is phytoene, which is then desaturated stepwise to yield the red pigment lycopene [22]. Lycopene can be considered the basic pigment that can be transformed, through different pathways, in cyclic carotenes and xanthophylls [23]. Carotenoids produced in plant chloroplasts are C40 or C40-derived carotenoid molecules, while in prokaryotic bacteria, C45 and most of the C30 and C50 carotenoids are produced, the rest of the identified forms are from archaea species [24]. A schematic presentation of the structure of various carotenoids is shown in Figure 1.

The most abundant, and the most important, among more than 1000 naturally occurring carotenoids, for application in the food and biomedical industry, are β-carotene, lycopene, lutein, and zeaxanthin. However, while some carotenoids (such as α-, β-, γ-carotene, and α-, β-cryptoxanthin) may be metabolized by humans into retinol (vitamin A), others (such as lutein, zeaxanthin, lycopene) are unrelated to any vitamin A activity in humans [25]. The physicochemical properties of carotenoids (such as color, chemical reactivity, molecular shape, light absorbing intensity, and antioxidant activity) depend on the length of the polyene chain (conjugated, delocalized π-electron system) [26]. The most important role of carotenoids is in the process of photosynthesis in plants and other photosynthetic organisms. Apart from chlorophylls that play the most important roles in light-depending reactions of photosynthesis, other important pigments in plants are carotenoids, betalains, and anthocyanins. Carotenoids are components of the photosystems I and II, together with chlorophyll, that have the role of accessory light-harvesting pigments, as well as photoprotective functions and appearance as precursors for biosynthesis of abscisic acid (ABA) and strigolactone [27,28]. Carotenoids that take part in photosynthesis are located in the chloroplast thylakoid membranes connected with the reaction centers and antenna complex of the photosystems I and II and are present in pigment protein complexes [29]. The role of carotenoids in photosynthesis is to absorb light at the wavelengths that chlorophyll molecules cannot, in the range of 460 and 550 nm, initiating the primary photochemical events of photosynthesis. Among more than 1000 naturally occurring carotenoids, only about 50 have a role in light-harvesting in photosynthesis [30]. As well as having a role in light harvesting, in chloroplasts, carotenoids have a photoprotective role under conditions of excess light [31], and due to their ability to scavenge ROS, carotenoids are involved in plants’ response to environmental stress. Carotenoids are oxidized by ROS and singlet oxygen, which are produced under stress conditions, to different aldehydes, ketones, endoperoxides, and lactones, which are very reactive electrophile compounds that can induce changes in gene expression and lead to responses to stress conditions [32].

Carotenoids occur in a variety of shapes and sizes, including Z and all-E isomers, and they have varying biological activities. While Z-isomers are bent, all-E isomers are linear molecules. Z-isomers often have a far reduced tendency to crystallize or aggregate. As a result, they have lower melting points and can be absorbed, transported, and solubilized more easily than their all-E counterparts [33,34]. The length and rigidity of the molecule, length of the conjugated double bonds, cyclized or uncyclized end groups, as well as the presence of polar substituents, are the most important characteristics of carotenoids which are responsible for their biological activity [33]. 

The most important use of carotenoids as a food ingredient is as antioxidants, pigments, or flavor modifiers [15]. In addition, carotenoids also have many different benefits for human health, but the most important is their antioxidant activity caused by their multiple conjugated double bonds, which can scavenge reactive oxygen species (ROS), absorb potentially damaging visible light, and inhibit the oxidation of lipids, proteins, and DNA, preventing many degenerative human diseases and disorders [15,35]. It is known that carotenoids exhibit different biological effects promoting health benefits in humans. They are a significant part of our diet and able to reduce the incidence of some chronic disorders, including inflammatory processes, cataracts, cardiovascular diseases, diabetes, muscular and neural tube defects, as well as Crohn’s, celiac, and other gastrointestinal diseases, as well as skin-related problems [36]. The prophylactic and immune-boosting effects of carotenoids are based on their high antioxidant capacity and ability to scavenge reactive oxygen species (ROS) due to their polyene backbone alleviating the unpaired electrons after radical quenching [37]. Moreover, it is known that carotenoids participate in different DNA anti-damage and repair systems and cell-nuclear signaling pathways [38]. Thanks to their proven health benefits and expressed antioxidant activity, carotenoids are today extensively used in the pharmaceutical, food, cosmetic, and nutraceutical industries [39]. However, although they present significant health benefits, their bioavailability is low. There are many reasons for this. First of all, they cannot be synthesized in humans, so they must be ingested through a diet. Secondly, they are highly insoluble in water due to their hydrophobic nature, which restricts their incorporation into foods and beverages. In addition, their highly unsaturated structure makes them highly susceptible to isomerization and oxidation, triggering their low storage stability and susceptibility to degradation when exposed to environmental stresses, such as oxygen, acidic pH, higher temperatures, light, enzymes, etc., which all result in color fading and loss of bioactive properties during processing and storage [21,35,40,41,42,43]. Overall, carotenes are more susceptible to thermal degradation than xanthophylls [42]. Furthermore, bioavailability of carotenoids is low even from fresh fruits and vegetables, their main dietary sources (10–20% for carotenes, and up to 40% for xanthophylls) [20]. The most important properties of the main carotenoids are summarized in Table 1.

As can be seen from the data presented in Table 1, general properties of carotenoids are their high antioxidant activity caused by the high number of conjugated double bonds, which enable them to protect from a wide range of different disorders and diseases connected to oxidation. At the same time, their structure makes them chemically very unstable, with a high tendency for degradation due to light, heat, and oxygen exposure. In addition, their relatively high MW combined with their lipophilic nature causes very low water solubility and, therefore, poor bioaccessibility.

In recent years, the market has displayed high demand for natural colorants and because of that, producers are oriented toward abandoning synthetic compounds. Among the most attractive natural colorants are carotenoids, besides betalain, chlorophyll, and antocyan. The main problem with natural carotenoids is their instability, which is reflected in oxidation in the presence of an oxidizing agent. Further, changes in pH, heating, and light exposure may change the natural structure of carotenoids as well epoxidan elongation of polyene chain, which contributes to changes in biological activity [50]. One of the ways to “save” the biological value of natural carotenoids is encapsulation (micro and nano). Therefore, not only during processing and storage of carotenoids, but also in order to increase the poor bioavailability of fresh carotenoids, it is of ultimate importance to protect them from degradation and to obtain targeted delivery. The bioavailability represents a key aspect of active compound actions at the target sites applied via different administration routes [51]. The concept of bioavailability includes two interconnected steps: Initial bioaccessibility followed by bioactivity. Bioaccessibility is a fraction of active compounds released from the food matrix, while bioactivity includes transport of active compounds toward the target sites [47]. The bioavailability of carotenoids is frequently reduced due to incomplete release from the food matrix, poor solubility, and potential degradation during digestion [21]. The encapsulation of β-carotenoid within Ca-alginate matrix improves its bioavailability and that phenomenon is pronounced for higher concentrations of alginate [52]. Obviously, there is a need to design protective and effective carotenoid delivery systems to overcome all these challenges. There are various techniques for this, and among them, encapsulation is one of the most promising. However, in order to be successful, appropriate encapsulating materials and methods must be used. Consequently, encapsulation of carotenoids within alginate-based matrices can improve bioavailability of carotenoids and protect them against digestive enzymes within the gastrointestinal tract [53]. For this purpose, alginate-based matrices should maintain mechanical and chemical stability under working and storage conditions.

## 3. Encapsulation

One of the major challenges is to create effective carriers for appropriate delivery of bioactive compounds to the human body and to reduce their deterioration or loss of beneficial properties, especially during processing caused by oxidation, degradation, evaporation, or thermal denaturation. One of the best strategies is to encapsulate unstable biomolecules either by coating active components into thin films or their entrapment within the matrix using different food-grade coating/entrapping materials [54]. However, it is still a problem to achieve high stability, high encapsulation efficiency, and sustained release of carotenoids within GIT at the same time [25,40,55,56]. The ideal encapsulating system for sensitive active compounds like carotenoids should reduce their reactivity under environmental conditions and help them to reach the desired location within the body for the release of carotenoids. In addition, carotenoid-loaded carriers should be biocompatible and hydrophilic in order to protect carotenoids and improve their stability [55].

There are generally three main approaches in encapsulation of carotenoids in order to improve their water solubility, storage, and working mechanical and chemical stability, bioavailability, and bioactivity, or/and to obtain their controlled and sustained release: Encapsulation into lipids, encapsulation into polymeric carriers, and their combination. The most commonly used delivery systems for carotenoids are oil-in-water emulsions (multilayer emulsions, multiple emulsions), polymer complexes, liposomes, micelle, Pickering emulsions, as well as starch and alginate-based hydrogels [20]. The best results in encapsulating carotenes were obtained by using micelles, emulsions, liposomes, and polymer complexes [21,57,58,59,60,61]. Among lipid-based encapsulating systems, emulsions are particularly suitable for encapsulation and protection of carotenoids. However, emulsion systems show limited ability to control the retention and release of the encapsulated materials [43].

Therefore, the best approach for liposoluble, heat-sensitive components is a combination of lipid-based and polymeric carriers. Proper selection of a suitable biopolymer for encapsulation depends on biopolymer functional properties, such as charge distribution and density, poly-dispersibility, ion type and ionic strength, MW, concentration, pH, solubility, and inter/intra molecular forces [25]. Different polysaccharides (i.e., starch, pectin, cyclodextrins, carrageenan, and alginate) have been frequently applied for the encapsulation of carotenoids. However, the obtained bioavailability was moderate, and in order to improve it, different proteins were used as coating agents [7,40]. Therefore, a combination of two or more biopolymers is desirable to achieve optimal functional properties through synergistic effects. Moreover, a combination of lipid/fatty acid biopolymers profoundly increases the bioavailability of encapsulated material and improves its stability and solubility [25].

**Figure 2:**
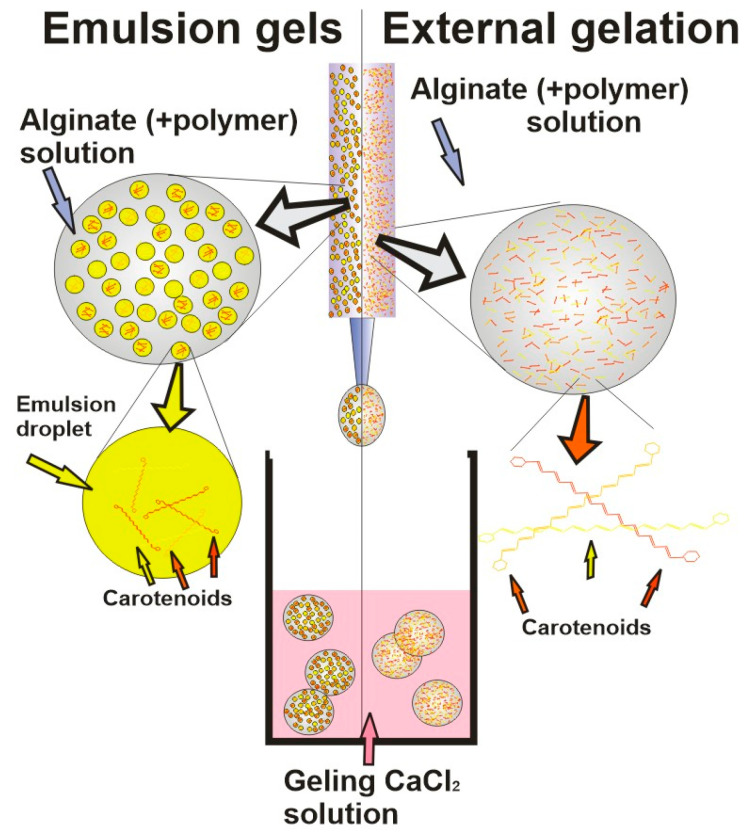
Emulsion gels and external gelation techniques schematic representation.

**Figure 3:**
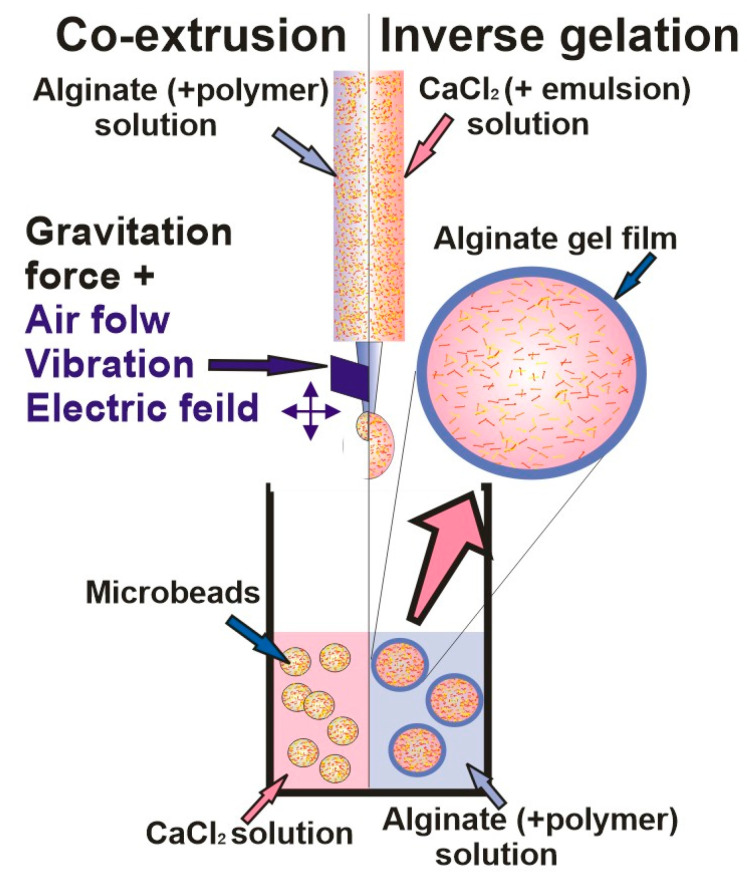
Co-extrusion and inverse gelation techniques schematic representation.

**Figure 4:**
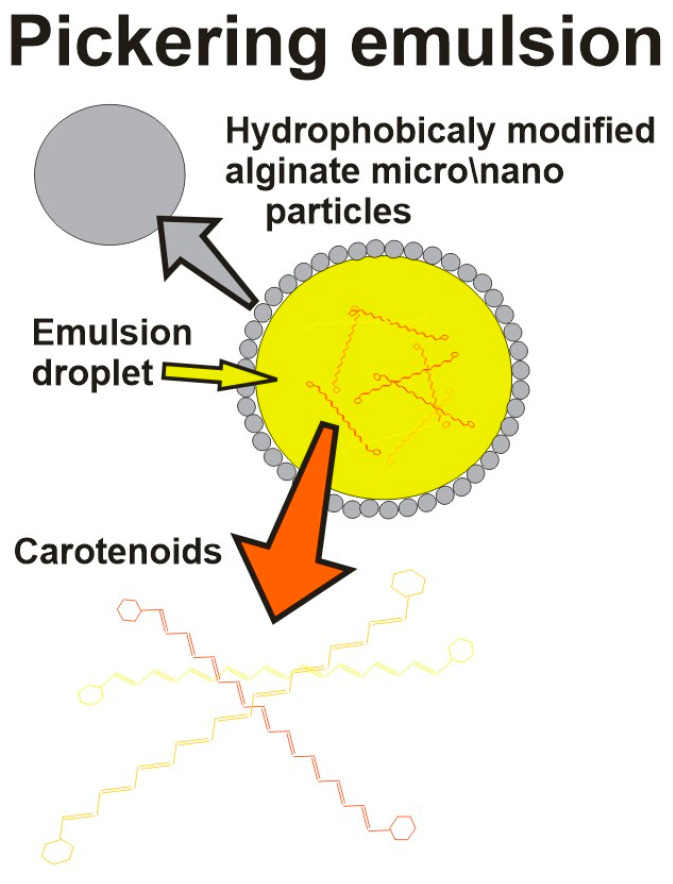
Pickering emulsion technique schematic representation.

Hydrogels based on alginate have been widely used carrier matrices for the encapsulation of hydrophobic and hydrophilic active compounds in various applications, such as food technology, biotechnology, and biomedical engineering [52,62]. Biocompatibility, low cost, mechanical stability under various experimental conditions accompanied by suitable rheological behavior and easy manipulation and processing of alginate into various shapes and dimensions have been recognized as a good choice for various applications [1]. However, while Ca-alginate gels offer very attractive, simple, and mild aqueous-based formation of gel (which is of the utmost importance for sensitive compounds) altogether with high levels of encapsulated carotenoids, there are also appropriate limitations (active component leakage, low mechanical strength, large pore size, and low cryo- and dehydro-capacities) that need to be overcome most commonly by mixing it with other biopolymers [7]. Namely, while those carriers mostly satisfy demands needed for encapsulation of hydrophilic active compounds, the carriers loaded by hydrophobic compounds need additional modifications. In those cases, hydrophilic alginate network blends with various polymers (i.e., proteins, complex lipids, polysaccharides, and low molecules, which have amphiphilic behavior) are frequently used for reduction of the carrier porosity and for the achievement of a controlled release of encapsulated materials [43,63]. Thus, polysaccharides like galactomannans may be used as wall materials to ensure and protect encapsulated bio compounds, while usage of gums (Gellan gum and Arabic gum) has shown improvements in gel intrinsic transport properties and stabilization of its microstructure, even during freezing and dehydration processes [64]. Table 2 summarizes the most important types of carotenoid encapsulating techniques, which are schematically shown in Figure 2, Figure 3 and Figure 4.

The effects that different alginate-based encapsulating systems have on some currently investigated carotenoids are summarized in Table 3.

As can be concluded from data presented in Table 3, alginate carriers in all cases improve the stability of carotenoids but at the same time reduce their bioaccessibility. The reduced bioaccessibility is caused mainly by prolonged release (hindered transport of active compounds through the gel matrix), and, in the case of micro and nanoparticles, this effect is either absent or, in some cases, even the opposite effect is observed, an increase in bioaccessibility. The same effects may also be observed for systems that use gel emulsion. Therefore, there are dual effects of encapsulation. On the one hand, the gel barrier offered by a matrix network hinders the rate of oxygen transfer and protects the bioactive from the action of light and aggressive compounds during passage through the digestive tract [74]. This is in agreement with studies that showed that the main factor for increasing stability (including photostability and oxidation stability) of encapsulated carotenoids is their isolation from water molecules in the polymer matrix [78]. On the other hand, the alginate gel matrix reduces carotenoids bioaccessibilty probably by increasing the macro-viscosity of the bulk aqueous phase and formatting hydrogel aggregates, entrapping lipophilic matter in the gastric phase [20].

## 4. Main Physical and Chemical Properties of Alginate Important for Gel Formation

It is necessary to discuss the main physical and chemical properties of alginates which influence their mechanical and chemical stability in order to understand better the benefits of the use of these materials as a carrier matrix for the encapsulation of carotenoids.

### 4.1. Sources

Alginate, a term encompassing anionic polysaccharides, primarily refers to sodium salt derivatives of alginic acid. These high-molecular-weight linear copolymers are predominantly produced by brown algae and certain bacteria [64,79]. While various seaweed species contribute to alginate production, the extraction of commercially available alginates predominantly relies on brown algae, where alginate constitutes up to 40% of the dry weight [70]. The composition of alginate can vary among seaweed species due to seasonal and growth conditions, as well as variations within different plant parts [80]. Alginate is primarily located in the cell walls and intercellular spaces, existing as an insoluble mixture of calcium, magnesium, potassium, and sodium salts, providing flexibility and strength to the plant and aiding growth in marine environments [81]. Additionally, alginate functions as a water reservoir, preventing dehydration when exposed to air, and is analogous to the roles of cellulose and pectin in terrestrial plants [82]. With a chain length of several thousand building units, alginate exhibits a partially rigid and partially flexible structure, similar to cellulose. Only two Gram-negative bacterial genera, Azotobacter and Pseudomonas, possess the capability to synthesize alginate as an exocellular polymeric material and bacterial biosynthesis can yield alginate with more defined chemical structures and physical properties (unlike seaweed-derived alginate), but commercially available alginates are exclusively sourced from algae, with molecular weights ranging from 32,000 to 400,000 g/mol [83,84].

### 4.2. Chemical Structure

Alginate, a naturally occurring polysaccharide, is a linear copolymer composed of mannuronic acid (M) and guluronic acid (G) units in their respective salt forms. The alginate chain exhibits a blockwise structure with β-D-mannuronic acid (M) and α-L-guluronic acid (G) connected through 1→4 glycosidic linkages. The presence of bulky carboxylic groups in M-M blocks leads to equatorial/equatorial glycosidic bonds, resulting in a flat ribbon-like chain conformation. In contrast, G-G blocks form axial/axial glycosidic bonds, leading to a buckled and rigid structure. The flexibility of the MG-block arises from the equatorial/axial glycosidic bond in M-G residues. Consequently, the rigidity of the chain blocks follows the order GG > MM > MG [1]. However, some analyses have shown that all blocks exhibit similar chain stiffness, regardless of their chemical composition [85]. The chemical and physical properties of alginate molecules are generally determined by the proportion and distribution of these three block types, including homopolymeric M blocks (MMMMM) and G blocks (GGGGG), as well as heteropolymeric blocks with an irregular blockwise arrangement of M and G residues (MGMGMG) [86]. The ratio of guluronate to mannuronate varies depending on the source of alginate, resulting in variations in the M and G contents and the length of each block among alginates derived from different sources [4].

### 4.3. Alginate Gelation

Alginates most remarkable characteristic is its ability to undergo gelation easily in the presence of polyvalent cations, particularly divalent cations like Ca^2+^. Moreover, alginate exhibits high water solubility, abundance, cost-effectiveness, biocompatibility, bioadhesion, biodegradability, non-toxicity, low immunogenicity, transparency, stability, and ease of handling. However, the limited stability of alginic acid necessitates its conversion into different salt forms to obtain stable, water-soluble, and functional commercial products [1].

Numerous studies have demonstrated that the chemical structure, molecular size, gelation kinetics, and gelling cation type profoundly affect various functional properties of the resulting alginate gel. These properties encompass porosity, swelling behavior, stability, biodegradability, gel strength, as well as immunological characteristics and biocompatibility [87].

In recent years, there has been a growing trend towards creating modified derivatives of alginic acid through different chemical and biochemical techniques, allowing precise control over the monosaccharide sequence, nature, location, and quantity of substituents [83].

#### 4.3.1. Gel Formation

Alginate is highly valuable in industrial and biomedical applications, mostly due to its gelation under mild conditions. The presence of hydrophilic groups (-OH, -CONH-, -CONH_2_- and -SO_3_H) contributes to alginate’s water-absorbing capacity and the formation of hydrogel structures [4]. 

The gelation of alginate is influenced by factors such as monomeric composition, block structure, molecular size, polymer concentration, and the presence of divalent ions [88]. Various methods, including ionic cross-linking, covalent cross-linking, thermal gelation, and cell cross-linking, can be used to prepare alginate hydrogels [84]. Among these methods, ionic cross-linking is particularly significant, as alginate exhibits a unique sol/gel transition in the presence of multivalent cations, such as Ca^2+^, Sr^2+^, and Ba^2+^, under mild temperature and pH conditions. This method is advantageous for applications involving sensitive biomolecules or living cells, as it employs non-toxic reactants like Ca^2+^ ions [2,83].

The gelation of Na-alginate with divalent cations, especially calcium chloride (CaCl_2_), is a rapid and irreversible process [89]. However, the rapid gelation caused by highly soluble salts like CaCl_2_ often leads to uncontrolled gelation, so to achieve uniform structures and optimal gel mechanical properties, it is crucial to control the gelation rate. This can be achieved by using less soluble salts such as calcium carbonate (CaCO_3_) or calcium sulfate (CaSO_4_) or by conducting gelation at lower temperatures to slow down the cross-linking process [84].

The choice of gelling cation significantly impacts the gelation process, and alginate exhibits varying affinity for different divalent ions. The affinity follows the order: Mg^2+^ << Mn^2+^ < Ca^2+^ < Sr^2+^ < Ba^2+^ < Cu^2+^ < Pb^2+^ [81]. This affinity is also influenced by alginate composition, including the M/G ratio, sequence, G-block length, and molecular weight. The G-blocks of alginate are mainly involved in intermolecular cross-linking with divalent cations, forming a chelated structure with calcium ions known as the egg-box model of cross-linking. Conversely, polymannuronate (MM) units exhibit typical polyelectrolyte characteristics of cation binding. The strength of binding between divalent ions and the three alginate fragments can be summarized as follows: GG blocks: Ba > Sr > Ca >> Mg; MM blocks: Ba > Sr >> Ca >> Mg; MG blocks: Ba >> Sr >> Ca >> Mg. Alginate gels exhibit different levels of rigidity, with decreasing rigidity order as follows: Pb > Cu, Ba > Sr > Cd > Ca > Ni > Zn > Co > Mn [1].

The mechanical properties of hydrogels are predominantly dependent on the polysaccharide composition. Specifically, the gel strength is associated with the length of G-blocks in the polymer rather than the overall content of guluronic acid residues in alginate. Research has indicated that polyguluronate (GG) units ranging from approximately 4 to 15 significantly impact Young’s modulus. Alginates with a high abundance of G residues exhibit increased ionic binding and mechanical rigidity. This effect is further enhanced by elongating the G-blocks and increasing the polymer’s molecular weight [4]. However, high-molecular-weight alginate solutions can become excessively viscous, which is undesirable for processing. To address this concern, a combination of high and low molecular weight alginate polymers can be employed to enhance the elastic modulus of gels while minimizing viscosity.

In addition to ionic gels, alginates can form acid gels under pH values below the pKa of the uronic acid residues. However, acid gel formation requires homopolymeric regions in alginate, where G-blocks play a critical role in stabilizing the gels. M-blocks also contribute to acid gel formation, which exhibits more of an equilibrium nature compared to ionic gels [1]. The stability of alginates to acid can be further improved by converting alginic acid to its propylene glycol ester. Acid gels have limited applications compared to ionic gels, except in certain pharmaceutical uses [4]. 

An alternative way to form gels is covalent cross-linking of alginate using various cross-linking reagents, such as chitosan, DEAE-dextran, amino-polyoxyethylene, poly(ethylene glycol)-diamines, poly-L-lysine, or proteins [1]. Another approach for covalent cross-linking is through photo-cross-linking, but some photo-cross-linking reactions may require the use of light sensitizers or release acid, which can be potentially harmful [4].

Alginate modified with cell adhesion ligands can cross-link with cells through weak and reversible ligand-receptor interactions, eliminating the need for additional cross-linking molecules. This gelation behavior is shear reversible and can be repeated multiple times since cross-linked structures are reestablished within a few minutes [2].

The strength, porosity, diffusion, alginate distribution, swelling-shrinking, and transparency are crucial technological properties of gels. By manipulating the formulation process, alginate gels with different firmness, softness, brittleness, or flexibility can be created [90]. High G alginates form robust and brittle gels with excellent heat stability due to their strong affinity for calcium, while high M alginates yield weaker but more tender and elastic gels with good freeze-thaw stability [1].

The mechanical strength of a gel also impacts its porosity and diffusion properties. Alginate gels rich in guluronic acid residues exhibit higher elastic moduli compared to gels with lower G residue content, and they also demonstrate faster diffusion rates [91]. This behavior can be attributed to the presence of long G blocks and short elastic segments in high-G gels, which establish a stiffer and static network in contrast to the more dynamic and entangled structure of low-G gels with longer elastic segments [90]. Furthermore, alginate gels exist as a combination of a solid and a solution, with the junction zones representing the solid state. Gels maintain their shape and withstand stress like solids, despite being composed of 99–99.5% water, with the remaining portion being alginate. Capillary forces contribute to the water-holding capacity of the gel, as most water molecules become physically trapped within the alginate matrix but can still migrate, which is vital for various applications such as cell immobilization [2].

To dissolve an alginate gel, sodium citrate or phosphate (or hexametaphosphate) solutions can be employed [92]. The gel structure can also be destabilized by sequestering calcium cations with soluble anions or substituting them with monovalent cations within the matrix [1].

##### 4.3.2. Physical Properties of Alginates

**Viscosity of Alginate Solution**

The viscosity of an alginate solution is influenced by several factors, including the extension and flexibility of the polysaccharide sample, average molecular weight (MW), temperature, solvent type, and ionic strength. Additionally, the structural properties of alginate, such as molecular mass and M/G ratio, impact solution viscosity and gel strength [4]. In terms of rheology, MW is a critical variable for the constitutive behavior of alginate solutions. Aqueous solutions of alginates display non-Newtonian, pseudo-plastic behavior characterized by the shear-thinning properties, where viscosity decreases with increasing shear rate. 

**Solubility**

The solubility of alginates in water is determined by three primary factors: Solvent pH, medium ionic strength, and the presence of gelling ions. Solution pH is a critical parameter in regulating alginate solubility, and when it decreases below the pKa of its constituent acids, phase separation or hydrogel formation occurs. Alginates rich in GG and MM residues have a higher propensity to undergo phase separation at acidic pH compared those in MG blocks [93].

**Biocompatibility**

Alginate falls under the generally regarded as safe (GRAS) classification by the FDA [87]. It is considered a biocompatible, non-toxic, and non-immunogenic biopolymer. Orally administered alginate is non-toxic and biodegradable, although it does not naturally degrade in mammals due to the absence of the alginase enzyme that can break down the polymer chains [2]. Commercially available alginates often have average MW exceeding the renal clearance threshold, indicating that they are not completely eliminated from the body even if the gel dissolves [94]. Partial oxidation of alginate chains improves their degradability under physiological conditions [84].

Although the biocompatibility of alginate has been extensively investigated in vitro and in vivo, the impact of alginate composition remains a topic of debate [4]. While high M content alginates have shown immunogenicity and induce approximately 10 times more cytokine production compared to high G alginates, other studies have observed minimal or no immune response to alginate implants [95,96]. This may be attributed to varying levels of purity in the examined alginate samples since highly purified alginate has demonstrated no significant foreign body reaction when implanted [2,84].

**Stability**

One of the most important conditions that the alginate hydrogel should satisfy is the chemical and mechanical stability of the hydrogel matrix under working and storage conditions. It means that it is necessary to keep the integrity of the hydrogel matrix under certain conditions and ensure the matrix disintegration under other conditions. Understanding the stability of alginate gels is essential due to its direct impact on long-term performance [97]. The stability of alginate is influenced by pH, temperature, and contaminants. Cleavage of the glycosidic linkages can occur in acidic, alkaline, and neutral environments in the presence of reducing compounds, temperature accelerates depolymerization reactions, while enzymatic degradation by lyase generates unsaturated compounds [1,98]. Handling alginate under neutral pH and minimizing heat exposure are necessary to avoid depolymerization [99]. Since the sterilization methods such as heat treatment, autoclaving, ethylene oxide treatment, and γ-irradiation can cause alginate degradation, autoclave sterilization should be avoided, and sterilization by filtration using 0.22-mm filters is recommended [1,100]. In addition, for calcium alginate hydrogels, the use of chelating agents, such as citrate, phosphate, lactate, and EDTA, should be avoided as they can displace the cross-linking ion from the network junctions, which leads to the gel disintegration. High concentrations of competing ions, such as Na^+^ in physiological saline solution (0.15 M NaCl), can also compromise network stability [84]. Ionic alginate gels have limited stability in physiological media due to ion exchange with monovalent ions, resulting in gel destabilization and rupture [90]. Strategies to enhance stability include chemical cross-linking or replacing calcium ions with stronger binding ions (i.e., Ba^2+^ or Sr^2+^) [1,101].

The mechanical stability of alginate hydrogel represents a product of cumulative effects of inter- and intra-chain interactions, which rely on micro-environmental conditions. Intra-chain interactions influence chain flexibility and, on that base, the chain conformations. Flexibility depends on the ratio between the chain’s persistent length and its contour length. When the persistent length is equal to or slightly larger than the contour length, the chains can be treated as semi-flexible [62]. The persistent length of alginate chains includes two contributions: (1) The intrinsic contribution caused by the chain’s chemical structure and (2) electrostatic contribution caused by the chain’s electric charge [102]. Chains made by long mannuronic blocks (M-blocks) are more flexible and form entangled network structures, while the chains made primarily by long guluronic blocks (G-blocks) are significantly stiffer [90]. The flexibility of alginate chains accompanied by the size and number density of egg-box-like junctional zones contribute to the matrix mechanical stability and the gel stiffness [62]. These junctional zones are a product of inter-chain interactions occurring in external ionic solutions, such as CaCl_2_. It is supposed that only the G-blocks of alginate chains rather than M-blocks take part in intermolecular cross-linking with Ca^2+^ cations [103]. Consequently, the M-G copolymer ratio accompanied by the concentration of alginate and the concentration of Ca^2+^ cations have an impact to the hydrogel stiffness and can vary in order to achieve the gel matrix with desirable mechanical properties [104,105]. The lower ratio corresponds to the higher value of the hydrogel Young’s modulus [82]. Ca-alginate hydrogel behaves as a viscoelastic solid [106].

The mechanical properties of the alginate hydrogel matrix depend on the cross-linker, M-G copolymer ratio, mesh size, as well as the working and storage conditions, and have been characterized by Young’s modulus, compression modulus, and shear modulus [107,108]. To enhance mechanical stability, high-G alginate with a high content of G units (>70%) and long G blocks (around 15 units) can be utilized, along with partial substitution of calcium ions with Ba^2+^. These factors contribute to improved mechanical and swelling stability of alginate beads. Alginate also forms strong complexes with polycations [79,90]. Covalent cross-linking in addition to physical (ion-induced) cross-links is another strategy to stabilize alginate gels. Various chemical techniques, including covalent grafting of alginate with synthetic polymers, combination of covalent and ionic cross-links, and direct reticulation of poly(l-lysine) on alginate, have been explored [1]. Enzymatic methods incorporating long alternating sequences into the polymer result in highly stable alginate gel beads that do not swell upon exposure to a saline solution [109].

In the case of oral administration, it is necessary to provide a chemically stabile matrix, resistant to attacks of gastric enzymes, such as pepsin, under acidic conditions and to ensure transport of active components toward the low intestine where the enzymatic disintegration of the matrix under a neutral condition should take place [51]. Ca-alginate hydrogels have been proposed in order to improve bioavailability, thermal stability, and biological activity of active compounds under simulated gastrointestinal conditions [52]. Mannuronic acid is more flexible and more reactive with pepsin than guluronic acid [110]. The alginate carriers also have to keep their integrity under storage conditions, which is essential for their application. The storage can be done in the form of dry powders or solutions. The stability of β-carotene loaded alginate carriers with 0.5% alginate prevented the degradation of β-carotene 1.5 times more efficiently than 1% beads at 25, 35, and 45 °C in darkness for 32 days which is in accordance with the fact that an increase in the concentration of alginate results in the formation of a more inhomogeneous gel structure for the same concentration of Ca^2+^ ions [6].

##### 4.3.3. Chemical Modifications

The integration of diverse chemical and biochemical techniques offers significant potential for the creation of modified derivatives of alginic acid with precise control over the sequence of monosaccharides, as well as the nature, location, and quantity of substituents. This capability enables the improvement of alginate derivative properties, including degradation, mechanical strength, and cellular interactions, which are of paramount importance. Additional properties that can be tailored include solubility, hydrophobicity, affinity for specific proteins, and others. The derivatization of alginate represents a convenient approach to enhance existing properties or introduce entirely new properties. Chemical modification serves as a tool to improve the ionic gel strength, increase its hydrophobicity, enhance biodegradation, and introduce anticoagulant properties and/or chemical/biochemical anchors for cell interactions. Consequently, the ability to chemically modify alginate holds tremendous potential for tailoring alginate materials for specific applications and creating next-generation biomaterials with enhanced or novel properties [83].

Alginate possesses abundant hydroxyl and carboxyl groups along the polymer chain backbone, thus providing two types of functional groups that can be modified to alter the characteristics relative to the parent compound. Alginates can be modified at the two secondary hydroxyl positions (C2 and C3) or the one carboxyl position (C6). The reactivity difference between hydroxyl and carboxyl groups allows for selective modification of either group. On the other side, the selective modification of C2 and C3 hydroxyl groups poses a challenging task due to their minor reactivity differences. Furthermore, it is possible to control the reaction in terms of selectively modifying M or G residues [83].

Through the functionalization of available hydroxyl and carboxyl groups, alginate properties such as solubility, hydrophobicity, and physicochemical and biological characteristics can be modified [1]. Chemical derivatization of the polysaccharide backbone enables enhanced hydroxyapatite nucleation and growth, heparin-like anticoagulant properties, improved cell surface interactions, degradability, and optimization of the hydrophobic-hydrophilic balance for optimal drug release [4]. Various chemical modifications of alginate have been achieved using different techniques, including oxidation, sulfation, esterification, amidation, and grafting methods.

The oxidation of sodium alginate can be accomplished by using sodium metaperiodate, which converts the secondary hydroxyl groups to aldehyde groups. This oxidation process increases the reactivity of alginate hydroxyl groups and allows for greater rotational freedom of molecules. Notably, the carboxylate groups remain unaffected, enabling the modified alginate to retain its ability to form ionic gels. Partial oxidation of alginates can be advantageous for biomedical applications, as they exhibit faster degradation in aqueous media compared to unmodified alginates, making them suitable for controlled delivery [4]. Additionally, oxidized alginates are more amenable to further modifications. However, the oxidation step may lead to a significant reduction in the viscosity of oxidized alginate due to MW degradation and chain conformational changes, which can lead to the formation of softer gel. The degradation of alginate, which results in a decrease in the MW during oxidation, depends on the sequence and distribution of M and G residues, with longer G block sequences degrading more rapidly than shorter MG blocks.

Alginates, previously oxidized with aldehyde groups, can be further modified through reductive amination, which involves their reaction with alkyl amines using appropriate reducing agents. This method has been employed to prepare alginate-derived polymeric surfactants. The addition of long alkyl chains to alginates imparts amphiphilic characteristics, such as lower surface tension, solubilization of solid azobenzene, and adsorption of heavy metals [1,111].

Additionally, alginates can undergo esterification reactions to introduce various substituents. Esterification can be achieved by reacting alginates with carboxylic acids or acid chlorides. This modification alters the hydrophobicity and solubility of alginate derivatives. For example, the introduction of long-chain alkyl esters can enhance the hydrophobicity of alginate, making it more suitable for applications such as drug encapsulation or tissue engineering scaffolds with controlled drug release properties [2,97].

Amidation is another method used for alginate modification, involving the reaction of carboxylic acid groups with amines or amino acids. This modification can introduce functional groups, such as amino or peptide moieties, which can facilitate specific interactions with biomolecules or cells. Amidation can also enhance the mechanical strength of alginate gels, making them more suitable for applications requiring structural support [2,112].

Sulfation is a chemical modification technique that involves the introduction of sulfate groups onto the alginate backbone. Sulfated alginates exhibit enhanced biological activities, such as increased anticoagulant properties or improved interactions with growth factors and proteins. Sulfation can be achieved by reacting alginates with sulfuric acid or sulfating agents [83].

Grafting methods involve attaching polymer chains or other functional groups onto the alginate backbone. This can be achieved through radical polymerization or coupling reactions. Grafting can introduce a wide range of functionalities, depending on the chosen grafting polymer or functional group, enabling the customization of alginate properties for specific applications [1].

It is important to note that the choice of modification method depends on the desired properties and applications of the modified alginate. Different modification techniques offer distinct advantages and limitations in terms of reaction conditions, selectivity, scalability, and stability of the modified alginate.

In summary, the chemical derivatization of alginate allows for the precise control and customization of its properties. Through modifications such as oxidation, esterification, amidation, sulfation, and grafting, the solubility, hydrophobicity, degradation rate, mechanical strength, and biocompatibility of alginate derivatives can be tailored. These modified alginate materials find applications in drug delivery, tissue engineering, wound healing, and various biomedical and industrial fields. The chemically altered structural changes of alginates have been considered by Raman spectroscopy as one of the most powerful techniques.

## 5. Raman Spectroscopy and Other Methods in Alginate and Carotenoids Analysis

Raman spectroscopy (RS) is an analytical method that uses coherent laser light to induce vibrations of molecule bonds. Generally, the Raman effect (or Raman scattering) represents the phenomenon of photons scattering by interaction between laser light (incident light) of a specific wavelength and a sample. The intensity of Raman lines is 0.001% of the intensity of the incident light (in the best case) and, as a consequence, the detection of scattering light is a difficult task. An exception is the Raman resonant spectra whose bands are of much higher intensity.

Raman spectroscopy/microscopy is a non-destructive spectroscopic analytical technique that can detect vibrational, rotational, or other low-frequency excitations from the observed system. In the case of single molecules, it is possible to detect vibrations able to change their polarizability. The characteristics of Raman spectra depend on the mass of atoms, the geometrical arrangements in the molecule, and the intensity of the forces connecting the atoms. The coupling of RS with microscopy techniques enabled a more precise structural and morphological analysis of different samples, providing further applicability in chemistry, physics, material science, medicine, food science and technology, etc.

The main advantages of RS are:

- Non or small preparative technique
- Small amount of sample
- Non-destructive
- Not interfered by water
- Spectra is acquired within a second
- Fast
- Relatively simple

Raman spectroscopy is already used for the determination of the alginate structure. The content of the uronic acids and their distribution in blocks varied with species and tissue types, and no relationship between M/G values and block composition in alginic acids was found. The information about the ratio between these two components can be obtained by RS. Bands at 1412, 1090, 955, and 708 cm^−1^ increase with increasing M/G ratio and the intensity of the bands at 1313, 1232, 884, and 806 cm^−1^ decreases with increasing M/G ratio [113]. 

There are a lot of different approaches to the application of RS in alginate analysis. Some authors propose 1064 nm lasers, while on the other hand, the 785 and 532 nm lasers have also shown good performance in alginate structure analysis (Table 4).

The hetero- and homopolymeric portions of alginic acid can be distinguished using SERS by characteristic bands [103]. In the fingerprint region, all the poly-D-mannuronate samples represent a band around 946 cm^−1^ assigned to C–O stretching, and C–C–H and C–O–H deformation vibrations, whereas the band at 863 cm^−1^ is assigned to deformation vibration of β-C1 –H group, and one at 799–788 cm^−1^ due to the contributions of various vibration modes. Poly-L-guluronate spectra show three characteristic bands: (a) At 928–913 cm^−1^, possibly assigned to the symmetric stretching vibration of C–O–C group, (b) at 890–889 cm^−1^ due to C–C–H, skeletal C–C, and C–O vibrations, and (c) at 797 cm^−1^ assigned to α C1 –H deformation vibration. The heteropolymeric fractions present two characteristic bands in the region, 730 cm^−1^ is more intense due to ring breathing vibration mode (Table 4).

The heterogeneity of biological macromolecules, such as alginates, because of their sequence, molecular weight, conformation, the small Raman cross-section, and the abundance of functional groups, result in significant band shifts, which further complicate the analysis using TERS. The Raman frequencies in the TERS spectra of biopolymers do not always correspond to band locations in the typical Raman spectrum of the bulk material [104].

The use of RS and chemometrics provides a quick, non-destructive, and reliable quantitative approach to determining the monomer composition (M/G ratio) of commercial alginate powders. The results showed a high correlation coefficient (0.97) while the prediction error (RMSEP) was 0.08. Comped to IR spectroscopy, RS displayed more reliable results [113]. 

Some characteristic Raman bands of alginate compounds are represented in Table 5.

It is already shown that RS is very convenient for the analysis of carotenoid-rich products without any special pre-preparatory sample procedure [118,119,120,121]. Generally, three main band positions of carotenoids are observed: (a) Around 1500–1550 cm^−1^ and (b) 1150–1170 cm^−1^ due to C=C and C-C stretching vibrations of polyene chain, as well as (c) around 1000–1020 cm^−1^ due to CH3 in plane rocking vibration. The location of the band(s) is well positioned. The wavenumber position of these bands is strongly correlated with the length of the carotenoid chain In general, carotenoids with 11, 9, 8, and 7 conjugated C-C bonds have their characteristic bands at approximately 1510, 1524, 1530, and 1536 cm^−1^, respectively [119]. 

Raman microspectroscopy can also be used as a tool for encapsulated carotenoid distribution assessment, as well as for multilayer complex analysis. According to the existing literature, there are scarce data on the application of RS in studies of encapsulated carotenoids. The combination of electrospinning with other technologies from the micro/nano encapsulation field in order to incorporate beta carotene into water-based food formulation avoids problems of its hydrophobic characteristics. They used Raman microspectroscopy to evaluate the distribution of beta carotene in this new product. For the β-carotene encapsulated into a three-layer complex which was made from different polysaccharides–xylan, chitooligosaccharides and fucoidan, and formation of the complex was confirmed by Raman spectroscopy [122]. However, there are no reports so far on the use of RS in studying the alginate-carotenoids systems, which is anticipated among the target priorities of our research group.

Besides RS, FT-IR (Fourier-transform infrared) spectroscopy can be used for analyses of nano- and microcapsulated materials.

FT-IR is a spectroscopic technique complementary to Raman spectroscopy, and it is also used for analyses of the structural composition of different samples. In FT-IR assessment of encapsulated particles, there are several bands which are observed. Bands at 3423.58 and 2925.67 cm^−1^ are the result of stretching vibration of the O-H and C-H bonds which is connected to sodium alginate structure. Moreover, the chitosan is characterized by band at 3423.42 cm^−1^, which is linked to the O–H stretching vibrations, whereas the bands at 1597.68 and 1654.42 cm^−1^ are attributed to the bending vibration of N-H and the stretching vibration of C=O, respectively. A common feature in chitosan is C-O stretching vibration, which is reflected in bands at 1258.15 and 1064.33 cm^−1^. Considering the beta carotene, absorption bands at 2925.57 and 2854.43 cm^−1^ are caused by the vibration of the hydrophobic group CH_2_ [53]_._

According to [123], the characteristic bands of C-H stretching vibration, COO− stretching vibration, and CH_2_ bending were observed at 2925, 1747, and 1466 cm^−1^, respectively. These bands were determined for encapsulated carotenoids isolated from the orange peel. 

Atomic Force Microsocpy (AMF) is also a technique that gave good results in studies of the encapsulated material. Moreover, information about particle and aggregate size can be obtained using AMF. AFM microscopy is an influential surface screening s technique used for micro/nanostructured coatings [124].

AFM is made up of a cantilever-mounted sharp tip with a diameter of between 10 and 20 nm. Si and Si_3_N_4_ are used in the micro-fabrication of AFM tips and cantilevers. The movement of the tip is detected by focusing a laser beam on a photodiode in response to interactions between the tip and the surface. The AFM platform allows the fully-integrated use of confocal Raman microscopy and can be performed with every laser source available by Raman spectrometer or could be provided by some other external illumination (e.g., solar simulator or other tunable or continuum source) [124,125].

## 6. Conclusions

In conclusion, alginate, and alginate hydrogels, due to their advantageous properties, have been widely used for the encapsulation of many bioactive components, among which are carotenoids. Their properties make their application a challenging task due to factors such as insolubility in water, susceptibility to degradation, and limited absorption in the human body. Encapsulation of carotenoids within suitable delivery systems, such as alginate-based carriers, offers a promising matrix to enhance their stability, solubility, and controlled release, thereby improving their bioavailability and preserving their beneficial properties. When selecting alginate as a functional gelling agent for applications such as encapsulation and controlled release systems, it is crucial to possess knowledge about the chemical and physical properties of alginate, its limitations, and its behavior when combined with other polymers. As has been concluded from reviewed published papers, all alginate-based systems considerably improve carotenoid stability, but only systems with micro- and nano-size, or emulsion-based, do not lack low carotenoid bioaccessibility. As a further way to improve alginate-based encapsulation system properties, other biopolymers may be added. Finally, the main techniques for evaluating the encapsulation of bioactive compounds were briefly considered. Therefore, this review may be a useful tool for researchers from different fields that deal with carotenoid encapsulation within alginate-based carriers for improving the encapsulation process of carotenoids, suggesting the best alternatives for those systems. In addition, a comprehensive cover of recent advances in this field is given.

## Figures and Tables

**Table 1:** The most important properties of the main carotenoids.

Component	Main Sources	Benefits	Limitations	Refs.
β-carotene	Carrots, sweet potatoes, tomatoes, apricots, squash, mangoes, yams, spinach, green peppers, green plants, and other fruits and vegetables.	Highest pro-vitamin A activity among the carotenoids, strong antioxidant that scavenges free radicals and reduces cancer, cataracts, infections, cardiovascular, and other chronic diseases, protects skin health, improves vision, strengthens immune system, and has immunomodulatory action.	Chemically unstable with high tendency for chemical degradation due to light, heat, and oxygen exposure due to large number of conjugated double bonds in its structure, high melting point, crystallinity at room temperature, extremely low water solubility, and very low (and variable) oral bioavailability (less than 10% from orally taken food).	[14,20,44,45]
Lycopene	Found in high concentrations in tomatoes, pink grapefruit, pink guava, papaya, and watermelon, also in apricots, barriers, plums, carrots, green peppers, red cabbage, passion fruit, and a large number of red-colored fruits, vegetables, and microorganisms.	Lycopene structure has a higher number of conjugated double bonds than other carotenoids, which makes it the most potent antioxidant among carotenoids (lycopene from watermelon had significantly higher antioxidant activity than from tomato). It possesses immunomodulatory and anti-inflammatory action, enhances immune system, prevents some types of cancer, cardiovascular, chronic, and degenerative diseases and hepatic fibrogenesis.	Chemically very unstable to oxygen, light, heat, and humidity exposure due to the presence of double bonds in its structure, easily oxidized and isomerized, destroyed at acidic pH, stable at basic pH, low water solubility induces low bioavailability, restricting its incorporation in different foods and beverages.	[3,14,44,46,47,48]
Lutein	Widely distributed in algae, fungi and bacteria, plants, especially in dark green leafy vegetables (broccoli, spinach, kale), orange-yellow fruits and vegetables (petals of the marigold flower, honeydew melon, mango, yellow corn, carrots, potatoes,) and egg yolks.	Lutein concentration is highest in the retina (~500 times higher than in other tissues), enhances eye health (proven retinal and macular protection against oxidative stress), maintains skin health, anti-diabetic, anti-obese, anti-cancer, anti-inflammatory antioxidant, antiatherogenic, anti-ageing, and immunomodulatory properties, prevents atherosclerosis, reduces prostate cancer risk.	Chemically unstable (sensitive to light, heat, pH, and oxidative stress, undergoes degradation due to interactions with other ingredients in the food matrix) crystalline at room temperature, very low water solubility, low bioavailability (in addition to the strongly hydrophobic nature of different nutrients, enzymes, and low pH in the GIT also decrease absorption), which all limit food and pharmacy applications.	[14,25,44,49]
Zeaxanthin	Egg, oranges, corn, honeydew melon, dark green leafy vegetables, bacteria.	Concentrated in macula prevents (as lutein) visual loss from age-related macular degeneration, prevents oxidation, cell and DNA damage, anti-ageing, prevents cardiovascular diseases, improves respiratory system and overall health.	Similar to lutein	[14,35,42]

**Table 2:** The most important types of carotenoid encapsulating techniques.

Encapsulating Method	Properties/Drawbacks	Refs.
**External gelation** - dropping polymer solution in cross-linking medium	- easy, low-cost procedure at extremely mild conditions, using a non-toxic and biocompatible polymer - produced beads could be used either directly or could be further processed - active compound retention, mechanical strength, and release rate could be managed by addition of other biopolymers - large beads provide better protection than the smaller ones but exhibit poor dispersion, while small beads have low microencapsulation efficiency - high encapsulation efficiencies and good carotenoid protection against adverse conditions	[65,66]
**Inverse gelation** - dropping cross-linking medium in polymer solution	- enables encapsulation of lipophilic active compounds or combination of compounds with different solubility into the same microparticles	[47,67]
**Emulsion gels** - emulsification followed by gelation for the preparation of oil droplets encapsulated in gel-like network structure - formed in two ways: First, adding emulsified oil droplets to the gel matrix under low oil phase conditions (emulsion-filled gel), second, emulsified droplets flocculated into network by other factors like high oil content (non-filled emulsion gel)	- used for encapsulation of lipophilic components - excellent storage stability and active substance encapsulation ability compared to emulsion - dual superior properties of emulsion and gel that combine advantages of emulsion encapsulation ability with texture of gel capable of resisting change of environment - good delivery systems for multiple functional ingredients that protect them from degradation and enable controlled release due to barrier effects of gel matrix and oil-water interface - while protein-based emulsion gel structures are easily disrupted during gastric digestion, polysaccharide-based emulsion gels (especially alginate) effectively maintain the gel structure during gastric digestion and release active substances slowly during intestinal digestion - sodium alginate synergistic protein emulsion gel considered an ideal delivery system for β- carotene - emulsion gel structure and physical properties may be optimized by regulating gel-forming conditions - proteins are used as emulsifiers to enhance encapsulation efficiency and storage stability of encapsulated nutrients - properties of alginate-based emulsion gels (i.e., morphology, structure, and gel strength) containing protein-coated droplets are affected by pH	[46,68,69]
**Co-extrusion microgels** - extruding a solution through a concentric vibrating nozzle (equal dimension droplets	- formed microgels can control the release of encapsulated materials - high porosity of alginate microgels may cause leakage of encapsulated materials - rapid degradation of encapsulated materials due to easy acid diffusion into microgels	[70]
**Pickering emulsions** - emulsions stabilized by particulate particles	- much longer sustained release of encapsulated lipophilic compounds than from traditional emulsions - improved stability of emulsion by outer solid layer	[71]

**Table 3:** Effects of alginate-based encapsulating systems on carotenoids properties.

Component	Encapsulation System	Benefits	Limitations	Refs.
β-carotene	Dried alginate beads	- improved stability for storage in dark and in reduced oxygen environment, particularly for lowest alginate content		[72]
	Alginate beads	- higher chemical stability within the GIT, particularly for higher alginate content - beads with lower alginate content increased storage stability (prevented degradation) more efficiently than those with higher content	- free lipid droplets had higher bioaccessibility than hydrogel beads	[6,51]
	Whey protein isolate (WPI)-alginate-chitosan capsules		- WPI-alginate-chitosan capsule coating inhibit bioaccessibility	[73]
	Alginate o/w emulsions	- enhanced chemical stability	- reduced bioaccessibility	[11]
	Whey proteins and HPMC-reinforced alginate hydrogels templated by emulsions	- incorporation of whey proteins and HPMC in alginate gel increases encapsulation efficiency compared to plain alginate particles	-	[60]
	Oil droplets in caseinate/alginate microparticles	- higher bioaccessibility and chemical stability	-	[19]
	Emulsion gels based on egg yolk granules and sodium alginate	- increased storage stability	- decreased bioaccessibility	[63]
	Zeinpropylene glycol alginate composite nanoparticles	- improved physicochemical stability and the sustained release	-	[44]
	Multilayer Alginate/Chitosan Gel Microspheres	- improved bioaccessibility and bioavailability	-	[52]
	Starch-alginate-gelatin emulsion-filled hydrogels	- improved stability, compared with free oil, increased shelf life	-	[74]
	Alginate-Pectin-Whey Protein Isolate Stabilized Emulsions	- high encapsulation efficiency - stabilized - emulsion had better thermal, physical, and chemical stability - low release in simulated gastric digestion, high release in simulated intestinal digestion	-	[75]
	Pickering emulsion stabilized by alginate-lysozyme nanoparticles	- enhanced stability against UV light		[76]
	Scallop gonad protein isolates stabilized emulsion in alginate beads	- greatly enhanced stability, prolonged release, and increased pH stability range	- higher bioaccessibility than emulsion-alginate beads	[77]
	Emulsion gel from egg yolk granules/sodium alginate bilayers emulsion	- emulsion gel structure denser at pH 4.0 than pH 7.0, which improves storage stability and chemical stability	- bioaccessibility decreased (sustained release)	[62]
Lycopene	Wet and dried alginate beads	- increased retention, especially for dried beads		[3]
	Internally and externally gelled alginate-based emulsion gels	- internal gelation produces emulsion gels with smaller oil droplets, and higher hardness, water-holding capacity, and freeze-thaw stability - internal gelation produces emulsion gels with a slightly higher bioaccessibility than those formed by external gelation.		[45]
	Alginate-based emulsion gels containing protein-coated droplets	- higher storage stability and bioaccesibility, faster release during in vitro digestion		[46]
	Alginate beads with added sugars and galactomannans	- lycopene release strongly influenced by beads composition		[59]
	Alginate beads with modified rice starch	- increased stability compared to emulsion and solution	- higher bioaccessibility for emulsions over beads	[40]
	inverse-gelated alginate microparticles	- particles prepared with low-viscosity alginate exhibited higher encapsulation efficiency and loading capacity		[47]
	Alginate beads with added sugars and galactomannans	- beads containing only alginate show severe lycopene decrease in all cases - alginate beads containing trehalose with β-cyclodextrin and Arabic gum showed high degradation protection regardless of freezing or drying method type - alginate beads and those supplemented with trehalose and vinal gum best preserved lycopene and minimized isomerization changes - alginate beads containing trehalose with guar gum showed high degradation protection regardless of freezing or drying method type - type of excipient influence level of protection and release		[7,59]]
Lutein	Chitosan-oleic acid- alginate nanocarrier	- increased solubility and bioavailability		[24]
	Alginate hydrogels	- retained release rate		[48]
	Alginate microspheres	- slower release at acidic conditions than at neutral intestinal conditions - radical scavenging activity of the microencapsulated lutein was higher than that of free lutein - significantly improved stability compared with free lutein		[54]

**Table 4:** Raman spectroscopy analytical methods used in alginate studies.

Type of Analysis	Laser Wavelength (nm)	Laser Power (mW)	The Aim of the Paper	References
FT-Raman	1064	200	Determination of Alginate Monomer Composition	[114]
Confocal Raman	785	0.03	Analysis of Hollow Silver Alginate Microspheres for Drug Delivery and Surface	[115]
SERS	633	0.2	Analysis of sodium alginates and their hetero- and homopolymeric fractions	[116]
SERS, TERS	532	0.2	Analysis of calcium alginate fibers and their network structures	[116,117]

**Table 5:** Characteristic Raman bands of alginate compounds.

Na Alginate + Ag	Ca Alginate + Ag	Na Alginate	Ca Alginate	Assignments	References
645–647 cm^−1^				Ring deformation	[116]
751–766 cm^−1^				Ring breathing	[116]
799–811 cm^−1^	812 cm^−1^	807 cm^−1^	816 cm^−1^	δ C–O–H, skeletal (ν C–C, ν C–O, δ C–C–H, δ C–C–O)	[117]
876–879 cm^−1^	831 cm^−1^	888 cm^−1^	888 cm^−1^		[117]
933 cm^−1^	870 cm^−1^	954 cm^−1^	959 cm^−1^		[117]
1132 cm^−1^	1104 cm^−1^	1098 cm^−1^	1088 cm^−1^	Glycosidic ring breathing mode	[117]
1281 cm^−1^	1266 cm^−1^	1300 cm^−1^	1300 cm^−1^	Carboxylate stretching vibration: Symmetric stretching or C–O single bond stretching vibration	[115,117]
1329, 1337, 1374, 1384, 1388 cm^−1^	1385, 1397 cm^−1^	1413 cm^−1^	1433 cm^−1^	Symmetric carboxylate stretching vibration	[117]
1358 cm^−1^				Aromatic stretching	[115]
1492, 1502, 1524, 1526, 1554, 1561, 1571 cm^−1^	1610 cm^−1^	1625 cm^−1^	1625 cm^−1^	Asymmetric carboxylate stretching vibration	[117]

## Data Availability

The data presented in this study are available on request from the corresponding author.
